# Association between multimorbidity, intensive care unit admission, and death in patients with COVID-19 in Brazil: a cross-section study, 2020

**DOI:** 10.1590/1516-3180.2022.0226.R1.21072022

**Published:** 2022-10-03

**Authors:** Jefferson Paixão Cardoso, Maria Inês Pardo Calazans, Aretha Lorena Fonseca Cantanhede Carneiro, Cayara Mattos Costa, Edna Luisa Oliveira Monteiro, Liliana Yanet Gómez Aristizábal, Juliana da Silva Oliveira, Alcione Miranda dos Santos

**Affiliations:** I PhD. Physiotherapist and Full Professor, Department of Health II, Núcleo de Estudos em Saúde da População (NESP), Universidade Estadual do Sudoeste da Bahia (UESB), Jequié (BA), Brazil.; II MSc. Physiotherapist and Doctoral Student, Postgraduate Program in Nursing and Health, Núcleo de Estudos em Saúde da População (NESP), Universidade Estadual do Sudoeste da Bahia (UESB), Jequié (BA), Brazil.; III MSc. Dental Surgeon, Postgraduate Program in Dentistry, Universidade Federal do Maranhão (UFMA), São Luís (MA), Brazil.; IV MSc. Dental Surgeon, Doctoral Student, Postgraduate Program in Dentistry, Universidade Federal do Maranhão (UFMA), São Luís (MA), Brazil.; V MSc. Dental Surgeon, Postgraduate Program in Dentistry, Universidade Federal do Maranhão (UFMA), São Luís (MA), Brazil.; VI PhD. Health Administrator, Postgraduate Program in Public Health, Universidade Federal do Maranhão (UFMA), São Luís (MA), Brazil.; VII PhD. Nurse and Full Professor, Department of Health II, Núcleo de Estudos em Saúde da População (NESP), Universidade Estadual do Sudoeste da Bahia, (UESB), Jequié (BA), Brazil.; VIII PhD. Statistician and Full Professor, Postgraduate Program in Public Health, Universidade Federal do Maranhão (UFMA), São Luís (MA), Brazil.

**Keywords:** Multimorbidity, Morbidity, COVID-19, Hospitalization, Death, Comorbidity, Coronavirus deaths in Brazil, COVID-19 prevalence studies, Hierarchical multiple logistic models, Intensive care unit

## Abstract

**BACKGROUND:**

Multimorbidity can influence intensive care unit (ICU) admissions and deaths due to coronavirus disease (COVID-19).

**OBJECTIVE:**

To analyze the association between multimorbidity, ICU admissions, and deaths due to COVID-19 in Brazil.

**DESIGN AND SETTING:**

This cross-sectional study was conducted using data from patients with severe acute respiratory syndrome (SARS) due to COVID-19 recorded in the Influenza Epidemiological Surveillance Information System (SIVEP-Gripe) in 2020.

**METHODS:**

Descriptive and stratified analyses of multimorbidity were performed based on sociodemographic, ventilatory support, and diagnostic variables. Poisson regression was used to estimate the prevalence ratios.

**RESULTS:**

We identified 671,593 cases of SARS caused by COVID-19, of which 62.4% had at least one morbidity. Multimorbidity was associated with male sex, age 60–70 and ≥ 80 years, brown and black skin color, elementary education and high school, ventilatory support, and altered radiologic exams. Moreover, all regions of the country and altered computed tomography due to COVID-19 or other diseases were associated with death; only the northeast region and higher education were associated with ICU admission.

**CONCLUSION:**

Our results showed an association between multimorbidity, ICU admission, and death in COVID-19 patients in Brazil.

## INTRODUCTION

The coronavirus disease (COVID-19), caused by severe acute respiratory syndrome coronavirus-2 (SARS-CoV-2), rapidly spread worldwide, causing approximately 185 million cases and more than 4 million deaths between December 31, 2019, and June 30, 2021.^1^

In Brazil, COVID-19 cases that progress to severe acute respiratory syndrome (SARS), leading to hospitalizations and deaths, are monitored using clinical samples analyzed in reference laboratories. Case notification is mandatory, and records are stored in the Influenza Epidemiological Surveillance Information System (SIVEP-Gripe) from the SARS Surveillance network, initially implemented to monitor the influenza epidemic in 2000.^2^

Since the emergence of COVID-19, scientific literature has addressed the virological characteristics of SARS-Cov-2 and clinical complications arising from its infection in different populations. Although severity is high in older individuals and males, some studies have shown a relationship between COVID-19 and pre-existing morbidities^3,4^ (e.g., cardiovascular diseases),^5–7^ which are associated with increased intensive care unit (ICU) admissions and deaths.

Studies have also shown an association between morbidity and COVID-19; however, only a few have investigated multimorbidity (i.e., the co-occurrence of two or more chronic diseases for a specific period^8^) as a factor predisposing patients to ICU admission and death.^5,9^

Brazil had the highest number of COVID-19 cases in Latin America and currently it also has a high prevalence of diabetes, hypertension, and cardiovascular diseases.^5–7^ Therefore, studies on association between multimorbidity, and ICU admissions and deaths due to COVID-19, are needed to provide basic knowledge for more complex studies establishing multicausality.^5–7^ Therefore, this study aimed to analyze the association between multimorbidity, ICU admission, and death due to COVID-19 in Brazil.

## METHODS

### Study design and data source

This cross-sectional study was conducted using data from hospitalized patients with SARS, reported in SIVEP-Gripe (base population) between February 20 and December 31, 2020. SIVEP-Gripe is a Brazilian epidemiological surveillance information system implemented in 2000 to monitor the influenza virus. However, during the H1N1 pandemic (2009), SARS surveillance was implemented in the Brazilian hospital network^2^ which became important for the notification of SARS cases during the COVID-19 pandemic.

We considered SARS in patients diagnosed with COVID-19 when they presented with flu-like syndrome followed by dyspnea, respiratory distress, persistent chest tightness, oxygen saturation < 95%, or cyanosis (i.e., bluish discoloration of lips or face).^2^ Moreover, cases should have been be reported in the SIVEP-Gripe, according to the Epidemiological Surveillance Guidance: Public Health Emergency of National Concern due to COVID-19.^2^ All patients with SARS due to COVID-19 were included (study population), except pregnant women, because pregnancy is a temporary condition that affects physiological functions independent of the disease^10^ and should be studied separately. Pregnant women represented 0.97% of the patients with SARS due to COVID-19 and were identified using Question 11 of the notification form.^2^

The database of SARS cases from 2020 was obtained from the openDataSus platform of the Brazilian Ministry of Health on May 3, 2021 (https://opendatasus.saude.gov.br/). We also obtained a dictionary of variables and SARS notification form. Unspecified SARS cases accounted for 37.3% of the total records.

### Variables

Outcome variables were ICU admission (yes or no), and death due to COVID-19, which were based on the progression of cases to “yes” (death due to COVID-19) or “no” (cure or death due to other causes) answers.

The independent variable, multimorbidity, was addressed using Question 36 (“Do you have risk factor or comorbidities?”) on the SIVEP-Gripe notification form, which had 14 answer options (puerperium, Down syndrome, asthma, diabetes mellitus, obesity, immunodeficiency or immunosuppression, cardiovascular, hematological, neurological, liver, kidney, or lung disease, among others). However, the puerperium option was not evaluated. We also identified other morbidities in option “others”. After identification and grouping, the following morbidities were included in the study: cancer, diabetes mellitus, dyslipidemias, obesity, systemic arterial hypertension, hypothyroidism, immunodeficiency or immunosuppression, and cerebrovascular, cardiac, hematologic, psychiatric, neurological, respiratory, liver, and kidney diseases. Multimorbidity was defined as a case (presence of at least two morbidities) and non-case (one morbidity). The number of morbidities (from one to five or more) was also included.

Independent variables were the following:

- Sociodemographic variables:
  - Sex (female or male);
  - Age (days, months, years). Patients were categorized into age groups (20–39, 40–59, 60–79, and ≥ 80 years) based on the age distribution according to chronic morbidities from the National Health Survey 2019.
  - Race or skin color (white or yellow, black, and brown). We excluded the indigenous category because it represented only 0.38% of the SARS cases due to COVID-19.
  - Educational level, categorized as no education, complete elementary education (1^st^ to 9^th^ year), high school (1^st^ to 3^rd^ year), or higher education
  - Brazilian regions (midwest, northeast, north, southeast, and south) were categorized based on data from the states of residence (including the Federal District) of patients.
- Ventilatory support and diagnostic variables:
  Invasive ventilatory support (yes, no)
  - Positive radiologic examinations for COVID-19, collected in six categories (normal, infiltrated, consolidated, mixed, other, or not performed) and dichotomized into normal and altered (infiltrated, consolidated, mixed, and other).
  - Computed tomography (CT) was categorized as negative or positive for COVID-19 or other diseases. We did not assess the “not performed” category for radiologic examinations and CT.

### Statistical analysis

R software 4.0.4 (R Foundation, Vienna, Austria)^11^ was used to analyze the data. The absolute and relative frequencies were calculated for each morbidity and outcome.

We calculated the number of morbidities and estimated the prevalence (P%), prevalence ratio (PR), and 95% confidence interval (95% CI) for ICU admission and death due to COVID-19.

The association between multimorbidity and outcomes was investigated using raw (number of morbidities) and stratified (multimorbidity) analyses, according to sociodemographic, ventilatory support, and diagnostic variables.

Hierarchical adjusted analysis, associated multimorbidity and sociodemographic, ventilatory support, and diagnostic variables, with ICU admission and death. Three blocks were considered: country region, sociodemographic, and support and diagnostic variables.

Poisson model with robust variance was used to estimate PR and 95% CI since outcomes of interest had prevalence of > 10%.^12^ We selected variables using bivariate analysis between outcomes and region, sociodemographic, ventilatory support, and diagnosis variables; P ≤ 0.20 was set as cutoff point for initial model selection. The model was adjusted to retain variables with the lowest Akaike information criterion values and theoretical criteria. We then assessed the influential point (i.e., absolute value of standardized errors > 3) and collinearity between predictor variables (i.e., positive variables with values > 10). The Hosmer-Lemeshow test determined the goodness of fit of the final model, considering a good fit when P ≥ 0.05.

### Ethical aspects

This study used anonymous information from the public domain. Thus, authorization for data collection and approval by the research ethics committee were not required.

## RESULTS

A total of 671,593 (59.7%) out of 1,121,601 hospitalized patients with SARS recorded in the SIVEP-Gripe in 2020 were diagnosed with COVID-19 (≥ 20 years and not pregnant). Of these, 216,055 patients were admitted to the ICU (38.1%) and 219,405 (35.7%) died. Moreover, 62.4% (419,425) of the patients with COVID-19 had at least one morbidity, and 97.0% with up to three morbidities were hospitalized due to SARS.

Table 1 shows the frequency distribution of morbidities according to ICU admission and mortality. The frequency of morbidities ranged from 34.1% (systemic arterial hypertension) to 47.1% (kidney diseases) in patients admitted to the ICU, and from 16.0% (hypothyroidism) to 62.8% (cancer) in those who died.

**Table 1:** Bivariate analysis between isolated morbidities, intensive care unit (ICU) admission, and deaths in patients hospitalized for COVID-19 in Brazil, 2020

Morbidities	ICU admissions			Deaths		
	n^a^	n^b^	%	n^a^	n^b^	%
Diabetes mellitus	29,798	10,498	35.2	31,841	11,257	35.4
Dyslipidemias	409	156	38.1	396	64	16.2
Systemic arterial hypertension	20,227	6,905	34.1	21,566	7,042	32.7
Hypothyroidism	1,839	637	34.6	1,808	290	16.0
Immunodeficiency	3,775	1,368	36.2	3,965	1,646	41.5
Obesity	11,696	5,202	44.5	11,871	2,959	24.9
Cancer	616	228	37.0	721	453	62.8
Stroke	249	108	43.4	276	164	59.4
Cardiac diseases	80,063	31,032	38.8	83,150	30,647	36.9
Hematologic diseases	881	307	34.8	903	359	39.8
Liver diseases	1,027	443	43.1	1,104	597	54.1
Neurological diseases	6,174	2,472	40.0	6,603	3,642	55.2
Mental health disorders	942	325	34.5	953	231	24.2
Kidney diseases	3,312	1,560	47.1	3,582	1,931	53.9
Respiratory diseases	9,035	3,103	34.3	9,283	2,755	29.7

a Total for occurrences of COVID-19;

b COVID-19 cases that progressed to ICU admission or death.

We observed that 29.5% (57,331) of the patients admitted to the ICU and 25.2% (57,359) of the patients who died had no morbidities. The prevalence and prevalence ratio of ICU admissions and deaths increased with an increase in number of morbidities (Table 2).

**Table 2:** Bivariate analysis between number of morbidities, intensive care unit (ICU) admission, and deaths in patients hospitalized for COVID-19 in Brazil, 2020

Number of morbidities	ICU admissions		
	n^a^	n^b^ (P%)	PR (95% CI)
1	170,043	64,344 (37.8)	1.00
2	130,825	57,419 (43.9)	1.16 (1.15; 1.17)
3	53,215	26,706 (50.2)	1.33 (1.31; 1.34)
4	14,940	8,035 (53.8)	1.42 (1.39; 1.45)
5 or more	3,864	2,220 (57.4)	1.52 (1.47; 1.57)
	**Deaths**		
	**n** **a**	**n** **b** **(P%)**	**PR (95% CI)**
1	178,022	64,037 (36.0)	1.00
2	135,662	59,778 (43.9)	1.22 (1.21; 1.23)
3	54,287	27,513 (50.7)	1.41 (1.39; 1.42)
4	15,110	8,539 (56.7)	1.57 (1.54; 1.59)
5 or more	3,917	2,379 (60.7)	1.68 (1.63; 1.74)

a Total for occurrences of COVID-19;

b COVID-19 cases that progressed to ICU admission or death.

P% = prevalence; PR = prevalence ratio; 95% CI = 95% confidence interval.

Stratified analysis indicated a higher prevalence of ICU admissions and deaths in patients with multimorbidity at all types of sociodemographic variables (Table 3).

**Table 3:** Stratified analysis between multimorbidity, intensive care unit (ICU) admission, and death in patients hospitalized due to COVID-19 according to sociodemographic characteristics in Brazil, 2020

Variable		MMB	ICU admission			Deaths		
			n^a^	P%	PR (95% CI)	n^a^	P%	PR (95% CI)
**Sex**								
	Female	No	26,070	35.5	1.00	26,420	34.2	1.00
		Yes	43,319	44.3	1.25 (1.23; 1.26)	45,138	44.7	1.30 (1.29; 1.32)
	Male	No	38,268	39.6	1.00	37,606	37.3	1.00
		Yes	51,056	48.6	1.23 (1.21; 1.24)	52,860	49.0	1.31 (1.30; 1.33)
**Age (years)**								
	20–39	No	5,516	33.5	1.00	2,828	16.7	1.00
		Yes	3,736	41.8	1.25 (1.20; 1.29)	2,405	26.7	1.60 (1.52; 1.68)
	40–59	No	18,345	33.6	1.00	12,089	21.5	1.00
		Yes	21,882	42.9	1.28 (1.26; 1.30)	16,776	32.4	1.50 (1.47; 1.53)
	60–79	No	28,596	40.2	1.00	30,664	41.1	1.00
		Yes	49,433	48.0	1.19 (1.18; 1.21)	51,961	48.9	1.19 (1.17; 1.20)
	≥ 80	No	11,887	42.7	1.00	18,456	61.0	1.00
		Yes	19,329	48.5	1.14 (1.12; 1.15)	26,867	64.2	1.05 (1.04; 1.06)
**Education**								
	No education	No	1,607	33.3	1.00	2,910	57.0	1.00
		Yes	2,597	40.1	1.20 (1.14; 1.26)	4,111	60.8	1.07 (1.03; 1.10)
	Elementary school	No	10,142	34.3	1.00	13,037	42.3	1.00
		Yes	17,835	43.9	1.28 (1.26; 1.30)	21,984	52.6	1.24 (1.22; 1.26)
	High school	No	6,307	33.1	1.00	5,793	29.4	1.00
		Yes	9,151	45.9	1.39 (1.35; 1.42)	9,038	44.4	1.51 (1.47; 1.55)
	Higher education	No	3,843	38.3	1.00	2,404	23.8	1.00
		Yes	4,896	49.7	1.30 (1.26; 1.34)	3,806	38.8	1.63 (1.56; 1.70)
**Race or skin color**								
	White/yellow	No	26,397	37.7	1.00	25,132	34.5	1.00
		Yes	40,987	46.2	1.23 (1.21; 1.24)	42,028	46.3	1.34 (1.33; 1.36)
	Brown	No	20,369	35.9	1.00	23,754	39.8	1.00
		Yes	28,505	45.1	1.25 (1.24; 1.27)	32,626	49.7	1.25 (1.23; 1.26)
	Black	No	2,872	35.8		3,390	40.0	
		Yes	4,920	45.9	1.28 (1.24; 1.33)	5,783	51.7	1.29 (1.25; 1.33)
**Brazilian region**								
	Midwest	No	5,871	37.3	1.00	5,091	31.0	1.00
		Yes	8,597	46.3	1.24 (1.21; 1.27)	8,118	43.1	1.39 (1.35; 1.43)
	Northeast	No	15,094	39.5	1.00	16,841	41.8	1.00
		Yes	23,157	48.3	1.22 (1.21; 1.24)	25,050	50.2	1.20 (1.18; 1.22)
	North	No	3,889	29.8	1.00	6,457	45.1	1.00
		Yes	4,427	38.8	1.31 (1.26; 1.35)	6,658	55.2	1.22 (1.19; 1.25)
	Southeast	No	34,198	38.9	1.00	31,140	34.0	1.00
		Yes	47,913	46.8	1.20 (1.19; 1.22)	48,076	45.7	1.34 (1.33; 1.36)
	South	No	5,288	35.1	1.00	4,502	29.4	1.00
		Yes	10,277	45.5	1.30 (1.26; 1.33)	10,098	44.1	1.49 (1.46; 1.54)

a Number of people affected by COVID-19.

MMB = multimorbidity; P% = prevalence; PR = prevalence ratio; 95% CI = confidence interval.

The prevalence of ICU admission (48.6%) and death (49.0%) were high in males with multimorbidity. Moreover, patients with multimorbidity aged 60–79 years and ≥ 80 years had 48.0% and 48.5% prevalence of ICU admission, respectively. Patients aged ≥ 80 years also had a high mortality rate (64.2%).

The prevalence of ICU admission was higher in patients with multimorbidity, with higher educational levels (49.7%) than in those with lower educational levels (40.1%). However, deaths were more frequent in patients with a lower educational level (60.8%) than in those with a higher educational level (38.8%). Black and brown patients presented with ICU admissions at 45.9 and 45.1%, respectively. They also presented a high prevalence of death (black patients, 51.7%; brown patients, 49.7%). The northeast region had a prevalence of 48.3% for ICU admissions, whereas the northern region had 55.2% of deaths (Table 3).

Regarding the associations between multimorbidity and outcomes according to support and diagnostic variables, the prevalence of ventilatory support was high in patients admitted to the ICU (52.7%) and those who died (51.1%). We also found that a high prevalence according to imaging tests; altered radiologic exams were associated with ICU admission (49.3%) and death (48.6%), while CT positivity for COVID-19 or other diseases was associated with ICU admission (50.3%) and death (41.5%) (Table 4).

**Table 4:** Stratified analysis between multimorbidity, intensive care unit (ICU) admissions, and deaths in hospitalized patients due to COVID-19 according to support and diagnostic variables in Brazil, 2020

Variables		MMB	ICU admissions			Deaths		
			n^a^	P%	PR (95% CI)	n^a^	P%	PR (95% CI)
**Ventilatory support**								
	No	No	6,662	17.1	1.00	6,672	17.5	1.00
		Yes	7,564	21.0	1.23 (1.19; 1.26)	8,789	25.0	1.43 (1.39; 1.47)
	Yes	No	52,886	44.8	1.00	46,429	40.5	1.00
		Yes	81,340	52.7	1.18 (1.17; 1.19)	76,960	51.1	1.26 (1.25; 1.27)
**Radiologic exams**								
	Normal	No	1,158	31.5	1.00	1,003	27.9	1.00
		Yes	1,524	38.4	1.22 (1.14; 1.30)	1,481	37.9	1.36 (1.27; 1.45)
	Altered	No	21,343	39.6	1.00	19,184	36.2	1.00
		Yes	34,541	49.3	1.25 (1.23; 1.26)	33,519	48.6	1.34 (1.32; 1.36)
**Tomography**								
	Negative	No	1,180	39.2	1.00	1,023	35.2	1.00
		Yes	2,041	45.3	1.16 (1.09; 1.22)	1,956	44.8	1.27 (1.20; 1.35)
	Positive for COVID-19 or other diseases	No	21,612	41.4	1.00	14,651	29.3	1.00
		Yes	33,781	50.3	1.21 (1.20; 1.23)	26,783	41.5	1.41 (1.39; 1.44)

a Number of patients with COVID-19.

MMB = multimorbidity; P% = prevalence; PR = prevalence ratio; 95% CI = 95% confidence interval.

The hierarchical adjusted analysis (Table 5) showed an association between multimorbidity and ICU admission and death after inclusion of variables (distal to proximal). These outcomes were also associated with male sex (ICU admission: PR = 1.15, 95% CI: 1.06–1.24; death: PR = 1.34, 95% CI: 1.24–1.46), 60–79 years (ICU admission: PR = 1.42, 95% CI: 1.21–1.66; death: PR = 2.96, 95% CI: 2.47–3.53), ≥ 80 years (ICU admission: PR = 1.55, 95% CI: 1.30–1.85; death: PR = 7.02, 95% CI: 5.76–8.56), brown color (ICU admission: PR = 1.14, 95% CI: 1.04–1.24; death: PR = 1.37; 95% CI: 1.23–1.51), black skin color (ICU admission: PR = 1.20, 95% CI: 1.03–1.41; death: PR = 1.77, 95% CI: 1.50–2.08), elementary education (ICU admission: PR = 1.34, 95% CI: 1.13–1.56; death: PR = 1.31, 95% CI: 1.12–1.55), high school (ICU admission: PR = 1.69, 95% CI: 1.43–1.99; death: PR = 1.38, 95% CI: 1.16–1.64), ventilatory support (ICU admission: PR = 5.50, 95% CI: 4.85–6.23; death: PR = 4.02, 95% CI: 3.53–4.58), and altered radiologic exams (ICU admission: PR = 1.63, 95% CI: 1.38–1.93; death: PR = 1.65, 95% CI: 1.38–1.96). Positive CT for COVID-19 or other diseases had a protective effect against death (PR = 0.65, 95% CI: 0.55–0.76).

**Table 5:** Hierarchical adjusted analysis for intensive care unit (ICU) admission and death in hospitalized patients due to COVID-19 according to independent variables in Brazil, 2020

Variables		ICU admissions			Deaths		
		Model 1^a^	Model 2^b^	Model 3^c^	Model 1^a^	Model 2^b^	Model 3^c^
		PR (95% CI)	PR^a^ (95% CI)^b^	PR^a^ (95% CI)^b^	PR^a^ (95% CI)^b^	PR^a^ (95% CI)^b^	PR^a^ (95% CI)^b^
**Multimorbidity**							
	No	1.00	1.00	1.00	1.00	1.00	1.00
	Yes	**1.42 (1.40; 1.44)**	**1.51 (1.48; 1.54)**	**1.46 (1.35; 1.57)**	**1.60 (1.57; 1.61)**	**1.58 (1.54; 1.62)**	**1.61 (1.48; 1.75)**
**Region**							
	Midwest	**1.06 (1.03; 1.09)**	0.95 (0.91; 1.01)	1.03 (0.87; 1.21)	0.99 (0.96; 1.03)	**1.11 (1.05; 1.17)**	**1.75 (1.46; 2.10)**
	Northeast	**1.15 (1.13; 1.18)**	**1.12 (1.07; 1.17)**	**1.59 (1.35; 1.86)**	**1.44 (1.40; 1.47)**	**1.57 (1.51; 1.64)**	**1.31 (1.10; 1.55)**
	North	**0.76 (0.74; 0.79)**	**0.73 (0.70; 0.77)**	0.86 (0.71; 1.03)	**1.66 (1.65; 1.77)**	**2.13 (2.01; 2.24)**	**2.39 (1.94; 2.93)**
	Southwest	**1.10 (1.08; 1.13)**	**1.10 (1.06; 1.13)**	1.08 (0.98; 1.20)	**1.12 (1.09; 1.15)**	**1.52 (1.47; 1.58)**	**1.22 (1.09; 1.36)**
	South	1.00	1.00	1.00	1.00	1.00	1.00
**Sex**							
	Female		1.00	1.00		1.00	1.00
	Male		**1.22 (1.19; 1.25)**	**1.15 (1.06; 1.24)**		**1.31 (1.28; 1.34)**	**1.34 (1.24; 1.46)**
**Age (years)**							
	20–39		1.00	1.00		1.00	1.00
	40–59		1.04 (0.99; 1.09)	1.07 (0.91; 1.26)		**1.34 (1.30; 1.46)**	1.18 (0.98; 1.41)
	60–79		**1.44 (1.38; 1.51)**	**1.42 (1.21; 1.66)**		**3.12 (2.96; 3.30)**	**2.96 (2.47; 3.53)**
	≥ 80		**1.55 (1.47; 1.63)**	**1.55 (1.30; 1.85)**		**6.90 (6.52; 7.31)**	**7.02 (5.76; 8.56)**
**Race or skin color**							
	White/Yellow		1.00	1.00		1.00	1.00
	Brown		0.99 (0.96; 1.02)	**1.14 (1.04; 1.24)**		**1.20 (1.17; 1.23)**	**1.37 (1.23; 1.51)**
	Black		1.02 (0.98; 1.07)	**1.20 (1.03; 1.41)**		**1.33 (1.27; 1.39)**	**1.77 (1.50; 2.08)**
**Education**							
	No education		1.00	1.00		1.00	1.00
	Elementary education		**1.15 (1.10; 1.20)**	**1.34 (1.14; 1.56)**		**0.88 (0.85;.0.93)**	**1.31 (1.12; 1.55)**
	High school		**1.26 (1.21; 1.33)**	**1.69 (1.43; 1.99)**		**0.77 (0.73; 0.81)**	**1.38 (1.16; 1.64)**
	Higher education		**1.48 (1.40; 1.56)**	**1.84 (1.53; 2.21)**		**0.59 (0.56; 0.63)**	1.15 (0.95; 1.40)
**Ventilatory support**							
	No			1.00			1.00
	Yes			**5.50 (4.85; 6.23)**			**4.02 (3.53; 4.58)**
**Radiologic exams**							
	Normal			1.00			1.00
	Altered			**1.63 (1.38; 1.93)**			**1.65 (1.38; 1.96)**
**Computed tomography**							
	Negative			1.00			1.00
	Positive for COVID-19 or other diseases			1.05 (0.90; 1.22)			**0.65 (0.55; 0.76)**

a Model 1: block 1 (region);

b Model 2: model 1 + block 2 (sex, age, race or skin color, and education);

c Model 3: model 2 + block 3 (ventilatory support, radiologic examinations, and computed tomography).

PR = prevalence ratio; 95% CI = 95% confidence interval.

We did not find associations between the three Brazilian regions and positive CT findings for COVID-19 or other diseases and ICU admission, or between higher education and death. Collinearity was not observed between variables. The most influential point was no lower than 0.005. Furthermore, the goodness-of-fit test indicated a good fit in both ICU admission (P = 0.358) and death (P = 0.105).

## DISCUSSION

We aimed to analyze the association between multimorbidity, ICU admission, and death due to COVID-19 in Brazil. We found associations between multimorbidity, male sex, black skin color, ventilatory support, and altered radiologic exams.

The high percentage of morbidities in the studied population was expected and corroborated the literature^13^ since individuals, with some morbidity and COVID-19, are more likely to be admitted to the ICU or they may expire.

The frequency of morbidities analyzed in this study (e.g., diabetes mellitus, systemic arterial hypertension, obesity, and cardiac diseases) was higher than those in the literature,^13,14^ even compared to a study conducted in the Brazilian population.^15^ We also obtained more robust results due to the size and national scope of the SIVEP-Gripe database, which is different from previous studies.^13–15^

The simultaneous effects of morbidities explain the increase in hospitalizations and deaths due to COVID-19. Therefore, assessing multimorbidity is important because some COVID-19 patients are expected to have other morbidities. Studies associated with metabolic syndrome and COVID-19 showed worsening of patients' conditions that led to ICU admission or death when two or three additional conditions (e.g., hyperglycemia, dyslipidemia, or arterial hypertension) were considered to classify this syndrome.^16,17^

The P% and PR of ICU admission and death due to COVID-19 increased with increase in the number of morbidities. This result was expected;^17^ however, the increase was significant in the presence of two or three morbidities. These data indicate a worse prognosis for patients with COVID-19 and multimorbidity, raising concerns for health services due to the high costs and increased demand of the health care personnel and technological support.

Analysis by age groups suggested that younger individuals were less affected by COVID-19 than adults and older individuals.^14,18^ We also found associations between age group and ICU admission or death in patients with multimorbidity. Age is an essential factor to assess the time to ICU admission or death due to COVID-19.^19^ Also, the time to ICU admission of older individuals may have been underreported since individuals belonging to this group are more likely to die before ICU admission.

This is the first study to report an increase in the P% of patients with multimorbidity admitted to the ICU with an increase in educational level. This result may be associated with better jobs, higher income, and better social living conditions in individuals with higher educational levels, suggesting availability of better healthcare. However, decrease in P% of deaths among individuals with higher education levels with multimorbidity was an inverse result. A study analyzing the socioeconomic aspects of COVID-19 lethality in Brazil showed that patients with higher education who had a more severe disease presented a lower prevalence of death than those with less education.^20^

Black and brown patients with multimorbidity have a high mortality rate. Another study also demonstrated that non-white patients, especially black patients, were more likely to develop severe conditions due to COVID-19, require ventilatory support in the ICU, and/or pass away.^21^

Regional disparities in socioeconomic development directly affected the number of COVID-19 cases. We observed that the northeast and north regions had the highest prevalence compared to other macro-regions of Brazil. Even considering that presence of, and access to specialized healthcare facilities for treating COVID-19 reduces the number of outcomes investigated in this study, access to health care must be considered in the most affected regions.

Complementary tests, such as radiologic and CT examinations, showed an relevant prevalence of ICU admission and death. Although these tests have good sensitivity, studies investigating complementary tests for COVID-19 have revealed low specificity compared with the reference diagnostic test (i.e., the reverse transcriptase real-time polymerase chain reaction). Nevertheless, some studies recommend using imaging tests to assess the extent of the disease and investigate possible complications,^22,23^ particularly in patients receiving ventilatory support in the ICU.^24^

Multivariate analysis indicated associations between male sex, age 60–70 and ≥ 80 years, black and brown skin color, elementary education, high school, ventilatory support, and radiological examinations. These findings corroborate with recent studies^25–27^ suggesting that sociodemographic factors are important predictors of ICU admission and death due to COVID-19.

This study had some limitations. Data may have been underreported, considering the lack of data regarding non-mandatory questions on the form. However, the sample size evaluated allowed us to demonstrate situations that were not revealed by other studies. Another limitation could be related to cases of SARS due to COVID-19 not detected by the Brazilian healthcare system, mainly those who did not have time to be treated in emergency care units or ambulances. Moreover, unreliable records may have influenced the results. Linking different databases may yield robust results. Finally, the SARS notification form did not inform whether deaths were caused during the disease or later due to post-disease complications. Similarly, the length of stay in the ICU may be a relevant factor in the assessment of cases.

In this study we highlight the assessment performed with the morbidities and outcomes, since it may be more expressive when considering isolated, dyad, and triad morbidities.

From the present study, we concluded that the prevalence of ICU admission and death was high in patients with morbidities, and that the increment in number of morbidities increased the prevalence and prevalence ratio of outcomes. An association between multimorbidity and ICU admissions due to COVID-19 was observed when adjusted for male sex, black and brown skin colors, age between 18 and 40 years, patients with some degree of education, use of ventilatory support, and altered radiological examinations. Regarding deaths due to COVID-19, multimorbidity was associated with male sex, black and brown skin colors, age ≥ 60 years, ventilatory support, altered radiologic exams, and CT findings indicating COVID-19 or other diseases.

Our findings may help train healthcare personnel to offer specialized care to patients with morbidities and COVID-19. Furthermore, we expect competent healthcare groups in the three spheres of the government to disseminate knowledge about multimorbidity and COVID-19 to reduce the spread of the disease and its impact on the healthcare system.
